# *mully*: An R Package to Create, Modify and Visualize Multilayered Graphs

**DOI:** 10.3390/genes9110519

**Published:** 2018-10-23

**Authors:** Zaynab Hammoud, Frank Kramer

**Affiliations:** 1 Department of Medical Statistics, University Medical Center Göttingen, Humboldtallee 32, 37073 Göttingen, Germany; 2 Institute of Computer Science, IT Infrastructure for Translational Medical Research, University of Augsburg, Universitätsstraße 6a, 86159, Augsburg, Germany; frank.kramer@informatik.uni-augsburg.de

**Keywords:** multilayered graphs, modelling in systems medicine, pathway modelling, pathway data integration, network visualization

## Abstract

The modelling of complex biological networks such as pathways has been a necessity for scientists over the last decades. The study of these networks also imposes a need to investigate different aspects of nodes or edges within the networks, or other biomedical knowledge related to it. Our aim is to provide a generic modelling framework to integrate multiple pathway types and further knowledge sources influencing these networks. This framework is defined by a multi-layered model allowing automatic network transformations and documentation. By providing a tool that generates this model, we aim to facilitate the data integration, boost the reproducibility and increase the interoperability between different sources and databases in the field of pathways. We present *mully* R package that allows the user to create, modify and visualize graphs with multi-layers. The package is implemented with features to specifically handle multilayered graphs.

## 1. Introduction

Network theory has been used for many years in the modelling and analysis of complex systems, as epidemiology, biology and biomedicine [1,2]. As the data evolves and become more heterogeneous and complex, monoplex networks become an oversimplification of the corresponding systems [1]. This imposes a need to go beyond traditional networks into a richer framework capable of hosting objects and relations of different scales and attributes [3,4], called Multilayered Network. These complex networks have contributed in many contexts and fields [5], although they have been rarely exploited in the investigation of biological networks, where their application seems very convenient [2].

In order to fill this gap, we present a multilayer framework that can be applicable in various domains, especially in the field of network modelling.

Our idea is to integrate pathways and their related knowledge into a multilayer model, where each layer represents one of their elements. The model offers a feature we call “Selective Inclusion of Knowledge”, as well as a collection of related knowledge into a single graph, like diseases and drugs.

The final aim is to provide a reproducible approach to integrate prior biomedical knowledge about networks in personalized medicine algorithms [1].

In this paper, we present an R software that we call *mully* (**mul**ti**l**a**y**ered graphs) that serves our objective by generating a generic model used for data integration. *mully* implements the multi-layer models within R and will subsequently be extended to parse various databases and further knowledge sources.

This paper consists of 3 main sections: in the first section, we give an overview on multilayered graphs and their implementation, a description of the model implemented in this package, as well as brief explanation about the implementation process of the package. The second is the Result section, where we highlight the most important features offered by the *mully* Package. Finally, in the third section we conclude.

## 2. Materials and Methods

### 2.1. Multilayered Graphs

**Figure 1:**
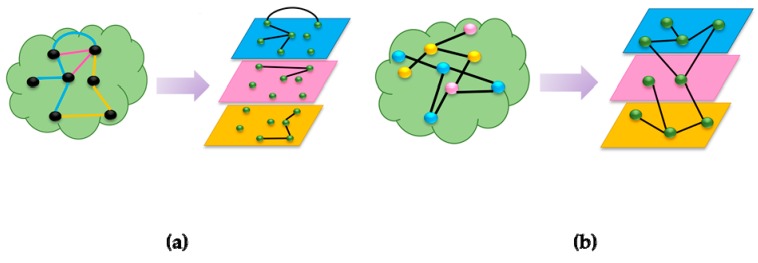
Two categories of multilayered graphs. (**a**) Edge-colored Graphs (ECG) (**b**) Node-colored Graphs (NCG).

Multilayered graphs are the new trend in Graph Theory used by a large number of scientists nowadays. They are employed in the modelling of big networks, with heterogeneous nodes (vertices) or relations (edges). Considering that this framework has many applications and in different fields, its interpretation and implementation depend on the subject that it’s serving. The main difference between these types is the criteria to link a node to a layer. In this context, two main types of networks can be distinguished: Node-colored graphs (NCGs) and edge-coloured graphs (ECGs) [1]. The following figures (Figure 1a,b) explain how to derive layers from regular graphs. On the left (Figure 1a) is an ECG, which is a graph with heterogeneous relations between the vertices. To transform this type of graphs into multilayered graphs, the nodes are replicated over all the layers, and each layer contains a subset of relative edges.

On the other hand, NCGs (Figure 1b) are graphs where nodes have aspects or types defined by colours. In order to build the multilayered graph from a NCG, the nodes are mapped to layers, leading to a network of networks, i.e., nodes having the same colours are grouped in the same layer. These graphs are usually layered-disjoint, i.e., the nodes can only be mapped to a single layer. 

**Figure 2:**
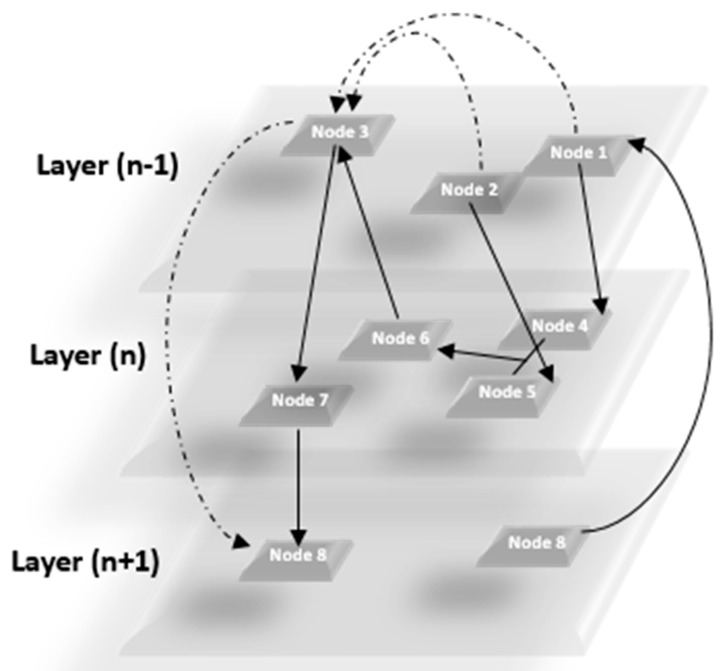
Generic Model of the Multilayered Graph implemented in our package.

The general model implemented in *mully* is a layered-disjoint NCG, and can be either directed or undirected. The following figure (Figure 2) gives an example of the generic model implemented in our package, which constitutes of n layers of nodes, connected with inter- and intra-layer edges.

### 2.2. Dependencies

The *mully* package is an R package that inherits the igraph object from the igraph R package [6] and adds additional information concerning the layers and other needed attributes. This package consists of a set of functions for nodes, edges, layers, graphs and visualization. The package is also set with a demo function that creates a sample graph used in order to try it.

It uses functions from other packages, for instance the 3D visualization is generated using the rgl R package [7], with some modifications applied concerning the layouting. It also refers to the RCX/ndexr package [8] to export the *mully* graph in Cytoscape Cyberinfrastructure Network Interchange Format (CX) [9] using its constructor.

## 3. Result and Features

**Figure 3:**
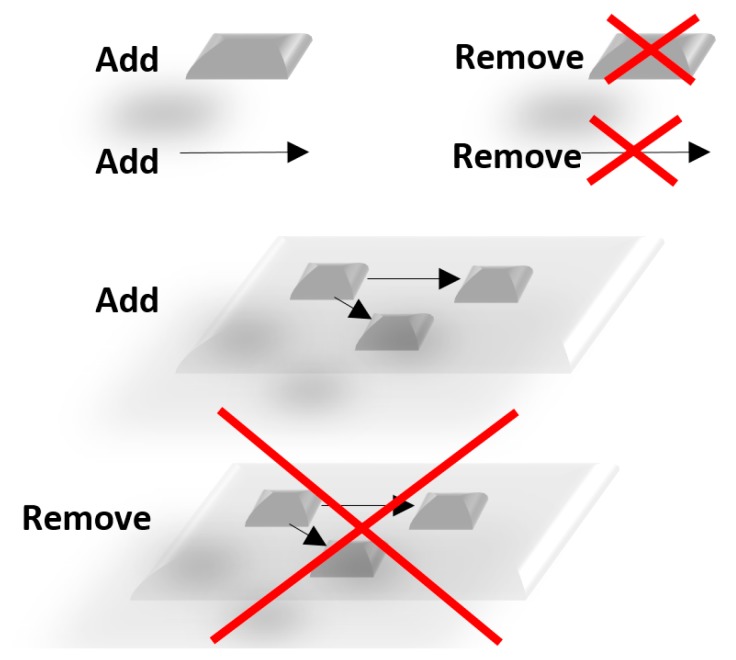
Standard Operations in *mully*.

The *mully* R package provides all the functionalities to work with graphs, which we call Standard Operations (Figure 3). In addition, we implemented special features to ease this work and the handling of big data import.

The main features are: transitivity, smart merging, undoing, visualization and converters.

### 3.1. Transitivity

**Figure 4:**
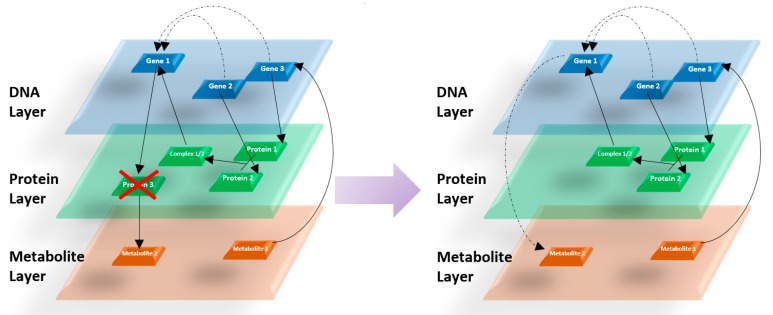
Addition of transitive edges after the deletion of a certain node.

One of the important functionalities offered by *mully* is the transitivity. When choosing to delete one or a set of nodes (a whole layer for example), the user can select to add the transitive edge before removing the incident ones. This feature is required in order to preserve the routes, especially when working with structured networks. In the following figure (Figure 4), we show an example of the impact of removing a node after choosing the transitive option.

### 3.2. Merging 

**Figure 5:**
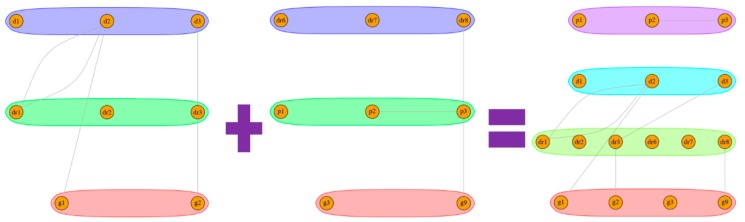
Layer Based Merging.

In order to provide an easy use of our package, we provide a smart merging function to create a single valid *mully* graph out of two inputs. The merge is based on the layers, i.e., the nodes from both input graphs are combined based on their assigned layers. This merging prevents the replication of data, for example in multiple sources, by monitoring the nodes and edges with same attributes (name, labels, etc.). Figure 5 shows a 2D visualization of two *mully* graphs, and the result of their merging.

### 3.3. Undoing

Using *mully*, the user is allowed to create multiple views from the same graph, where views are defined by the result of the application of a set of modifications to a graph. Since the views are derived from the same graph and data, we provide the undo function in order to help the user avoid repetitive actions. Undoing helps the user to fetch the original or previous states of the *mully* graph without having to recreate it. This feature is considered the most important in this package, since it serves one of our critical aims. Undoing helps the user to document all the steps that he followed to obtain the current version of the network that he possesses. The documentation of these steps of generating views will contribute to solving the reproducibility problem, observed mostly in the research field. It will guide researchers and scientists to obtain snapshots and fragments of networks and reproduce others generated in other research.

### 3.4. Visualization

**Figure 6:**
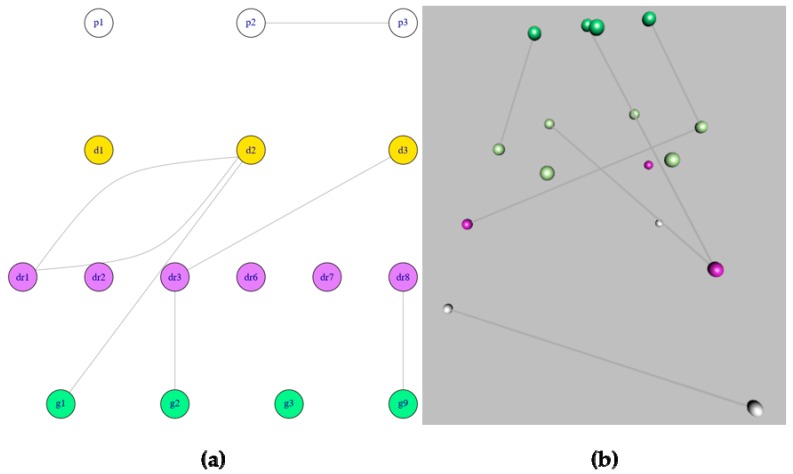
Visualization of the same network using the two different visualizers in *mully*. (**a**) 2D Visualization with a Scaled layout (**b**) 3D visualization.

The *mully* package also offers a visualizer for multilayered graphs. In this visualizer, we generate layouts based on the layers, by assigning different coordinates for the nodes, where the nodes belonging to the same layer are assigned coordinated in a range of similar numbers. The user can choose between two different layouts, the random and the scaled layouts. By choosing the random layout, the nodes within a layer are displayed on random points on the display screen, while choosing a scaled layout divides the layer area display between the nodes, always making sure to avoid any overlapping vertices in both cases. We also provide the user with two visualization options: 2D and 3D visualization. The 3D visualization is generated using the rgl R package [7] which is dedicated for interactive visualization. The visualization of the same graph is shown in Figure 6 in 2D Scaled layout and in 3D. 

### 3.5. Data Exchange

Cytoscape Cyberinfrastructure Network Interchange Format (CX) is a format for encoding network’s data, developed in conjunction with the Cytoscape group [9]. It is used as a standard for network interchange by Cytoscape [10], NDEx [11], and the services in the Cytoscape Infrastructure. As this format becomes one of the standards to exchange graphs, we believe that it is essential to include it in our package. The converter provided by *mully* aims to export *mully* graphs in a CX format by using the RCX/ndexr R package [8]. By exporting the graph as a CX object, the user can then import it and use it into other tools supporting the CX format such as Cytoscape.

In this package we also provide the feature to export the graph as a CSV file. In order to export the graph, three CSV files are generated; a file containing the information about nodes, a file containing the information about edges and another for layers. This export function can also contribute in the import of a *mully* object into *mully* or other packages and tools supporting multilayered models.

## 4. Discussion

Multilayered graphs are currently widely used by scientists for the manipulation of big heterogeneous networks, like Social Networks, Information Networks, Technological Networks and Biological Networks [12,13]. Despite this usage, the number of tools dedicated for these graphs are still insufficient such as Arena3D [14,15], muxViz [2], etc., while we have a rich collection of tools to handle and visualize big networks on a monoplex level, of which we mention Cytoscape [10], Cell Ilustrator [16], igraph [6], Cell Designer [17], RGraphviz [18], RCytoscape [19] and many others [20].

*mully* is an R package that allows the user to create, modify and visualize multilayered graphs. It is implemented with special features to ease the modification and the handling of graphs by the user. It is available for free usage on Github [21].

For us, *mully* will be the stepping stone to integrate different knowledge sources and provide a reproducible knowledge network to be integrated in systems medicine approaches.

## Availability

*mully* is a free, open-source R package available for usage via Github (https://github.com/frankkramer-lab/mully).
